# Community based programs to improve the oral health of Australian Indigenous adolescents: a systematic review and recommendations to guide future strategies

**DOI:** 10.1186/s12913-020-05247-w

**Published:** 2020-05-06

**Authors:** Josephine Gwynn, John Skinner, Yvonne Dimitropoulos, Angela Masoe, Boe Rambaldini, Vita Christie, Woosung Sohn, Kylie Gwynne

**Affiliations:** 1 Faculty of Medicine and Health, The University of Sydney, Sydney, Australia; 2 Poche Centre for Indigenous Health, The University of Sydney, Room 224 Edward Ford Building, Sydney, Australia; 3 NSW Ministry of Health Centre for Oral Health Strategy, 30 Christie Street, Wollstonecraft, Australia; 4 The University of Sydney School of Dentistry, 1 Mons Road, Westmead, Australia

**Keywords:** Indigenous, Adolescents, Community, Oral health, Oral health promotion

## Abstract

**Background:**

To review the international literature on community-based interventions aiming to improve the oral health of Indigenous adolescents and identify which demonstrate a positive impact.

**Methods:**

Data sources were MEDLINE, EMBASE, CINAHL, SCOPUS, the COCHRANE library and the Australian Indigenous Health*Info*Net. Articles were included where they: were published in English from 1990 onwards; described oral health outcomes for Indigenous adolescents aged 10 to 19 years; implemented a community based oral health intervention. The Quality Assessment Tool for Quantitative Studies from the Effective Public Health Practice Project was applied.

**Results:**

Nine studies met inclusion criteria; two rated strong in quality; only one study was conducted with an urban community; five reported moderate community engagement. Five intervention strategies were identified, and schools were the most common setting reported. Statistically significant improvements were described in eight studies with the most frequently reported outcome being change in decayed missing or filled teeth.

**Conclusions:**

Few good quality peer reviewed international studies of community-based oral health interventions which address the needs of Indigenous adolescents exist. Studies must include strong Indigenous community leadership and governance at all stages of the research, adopt participatory action-based research approaches, and are required in urban communities.

## Background

Internationally Indigenous peoples experienced thriving rich and diverse cultures over tens of thousands of years until the processes of colonisation severed connections to land and culture and devastated many communities [1]. This resulted in loss of land, spiritual and kinship disconnection and high burdens of poor physical and mental health [2] including poor oral health [3]. These burdens continue to exist and be compounded by ongoing socioeconomic, environmental and geographic factors. This paper uses the World Health Organisation (WHO) definition of Indigenous peoples as communities who live on their ancestral grounds, identify as part of a distinct cultural group and are descended from the first peoples of their land [1]. The term Aboriginal and Torres Strait Islander will be used when discussing the Indigenous people of Australia.

Indigenous peoples experience poorer oral health than their non-Indigenous counterparts and are less likely to receive timely dental care [4]. The social determinants of health are acknowledged as being at the centre of oral health inequalities experience by Indigenous peoples [3], however very few oral health studies explore these complex issues. Determinants of poor oral health for Indigenous peoples identified in the international literature in Brazil [5], Ecuador [6] and Canada [7] include: remoteness and community infrastructure such as access to electricity; consumption of processed foods; and racism.

Several studies describe the unacceptably high burden of poor oral health for Indigenous adolescents internationally. These include in the Indian Himalayas [8], Brazil [9, 10], Mexico [11], and Alaska [12] where high rates of dental pain (77%) tooth decay (71.3%) and pigmented lesions (47.6%) were reported. Maori children in New Zealand are more likely not to receive dental care than other children [13], and in Sri Lanka a high rate of oral cancer and potentially malignant oral disorders was found to exist among Indigenous Adolescents [14].

Several literature reviews have examined oral health interventions for Indigenous peoples internationally [3, 15–17]. These reviews found that successful interventions adopted community based participatory approaches that: are inherently collaborative and culturally appropriate; employed community workers in their delivery: and addressed the determinants of health [3, 15–17]. These reviews also report that: adopting an ‘ecological’ approach - namely a multi setting and multi strategy approach - to oral health prevention is promising; a consistent challenge faced at the intervention delivery level is sustained funding and; social and environmental contexts were significant barriers to good oral health [3, 15–17]. We found no literature reviews that examined the quality and effectiveness of oral health interventions specifically for Indigenous adolescents.

The Aboriginal and Torres Strait Islander population in Australia is young compared to the non-Aboriginal population (50% compared to 31% respectively between the ages 0 to 24 years) [18]. The health profile of Aboriginal and Torres Strait Islander adolescents differs considerably from their non-Aboriginal counterparts. Aboriginal and Torres Strait Islander adolescents experience several health conditions at much higher rates including poor oral health (10% higher) [19]. Studies report that 15% of Aboriginal and Torres Strait Islander adolescents aged 15–24 years have had their teeth extracted [19] and that those aged 14 to 15 years old have 4.1 permanent teeth on average affected by dental caries compared to only 2.4 for their non-Indigenous counterparts [20].

Adolescence is a complex period of great change including hormonally, sexually, physically, cognitively and socially [21]. Furthermore Indigenous adolescents experience additional challenges relating to the impact of marginalization, discrimination and poverty [22]. Given that health behaviours formed in this period can have lasting impacts on overall general health and well-being [23], including oral health, the need for culturally competent and effective interventions targeting this population is particularly important. This study aims to systematically examine the quality, community engagement (including leadership) and components of existing oral health interventions for Indigenous adolescents globally. The findings will contribute to the co-design of a community-based intervention which aims to improve the oral health of Australian Aboriginal and Torres Strait Islander adolescents.

## Methods

### Study selection process and eligibility criteria

In this systematic review, electronic databases were searched including: MEDLINE, EMBASE, CINAHL, SCOPUS, the COCHRANE library and Australian Indigenous Health*Info*Net. Simultaneously a hand search was conducted of the reference lists of key articles and the grey literature (World Catalogue, Google Scholar, OAlster, Australian Policy Online and National Library Australia (NLA@TROVE)).

The search terms included (‘dmft’ OR ‘dental caries’ OR ‘caries’ OR ‘dental care’ OR ‘oral hygiene’ OR ‘dental hygiene’ OR ‘fluoridation’ OR ‘fluoridating’ OR ‘oral cavity’ OR ‘tooth’ OR ‘gingiva’ OR oral health’ OR ‘periodontal disease’) AND (‘child’ OR ‘teenage*’ OR ‘adolescen*’) AND (‘indigenous’ OR ‘Aborig*’ OR ‘Torres Strait Islander’ OR ‘first nation’ OR ‘native’) AND (‘intervention’ OR ‘treatment’ OR ‘prevention’ OR ‘program’ OR ‘service’). This review included articles published only in English from 1990 onwards as the authors agreed that the relevance of data gathered prior to this would be limited by the policy and social contexts of those times which had yet to encompass the current post-colonial era [24].

Articles were included in the review if they met the following criteria: 1) described outcomes for Indigenous adolescents in the age range of 10 to 19 years [25] (or included young people of this age range); 2) quantitative measures that allowed for comparison between groups with and without interventions; and 3) described changes in one or more of the following measures: nutrition, tooth brushing behaviours, oral health knowledge, the number of decayed, missing and filled primary and/or permanent teeth (dmft/DMFT), dental caries, oral hygiene, gingivitis, and periodontal disease. Articles that described water fluoridation program implementation were also included if they met the other criteria. Articles were excluded if they reported solely clinical interventions or were whole of population studies which did not report by age range.

Article titles and abstracts were scanned and checked against inclusion criteria by JG and duplicate citations were removed. Those that met the inclusion/exclusion criteria were independently reviewed by JG, KG, JS and AM, and assessed for inclusion. Any disagreement about the eligibility of studies was then resolved by discussion until consensus was reached. The PRISMA checklist [26] of items to include when reporting a systematic review were followed and this review was registered with PROSPERO (number: CRD42018084673).

### Quality assessment

Articles were assessed for their quality using the Quality Assessment Tool for Quantitative Studies from the McMaster University Effective Public Health Practice Project (EPHPP) [27]. This includes six quality assessment domains (selection bias, study design, confounders, blinding, data collection methods and withdrawals and dropouts). An article was rated weak if it scored two or more weak component ratings, moderate if it scored one weak rating or strong if it scored no weak ratings. Articles were allocated for review by JG and reviewed by JS or JG in collaboration with AM. Any discrepancies in component ratings were resolved through discussion between the three reviewers.

### Community engagement intensity assessment

The principles of community engagement and governance as well as capacity building are critical in research with Aboriginal and Torres Strait Islander Australians [28, 29]. Therefore, these principles were assessed in all studies included in this review by JG and JS by considering four key features identified by JG from Australian guidelines for ethical conduct of research in Aboriginal and Torres Strait Islander communities [28, 30–33]. These features are collectively labelled as Community Engagement Intensity (CEI) and include 1): community governance of and engagement in research; 2) capacity building; 3) community-initiated research; and 4) feedback of results. Each study was assessed as possessing either a ‘light’ (≤ 1 feature), ‘moderate’ (2–3 features) or ‘strong’ (4 features) CEI. While this is not a validated measurement tool, it is a method of reporting the level of community engagement in a study and prior use of this scale is published elsewhere [34].

### Ecological approach assessment

An ‘Ecological’ model [35] adopts a multi-setting and multi-targeted approach to delivering an intervention [36] and is recommended when designing community based programs to address complex health issues, such as poor oral health, in Indigenous communities [15]. Therefore each study included in this review was given an ecological approach score (EAS) depending on its ecological complexity [35]. A score of 4 was given if the study included at least two strategies and implemented in more than 3 settings. Scores between 1 and 3 were given if the study included fewer strategy types and settings [35].

### Data extraction

General characteristics of the article, participants, interventions, study outcomes and measures were extracted by JG and AM using a purpose designed form.

## Results

### Study selection and characteristics

**Fig. 1:**
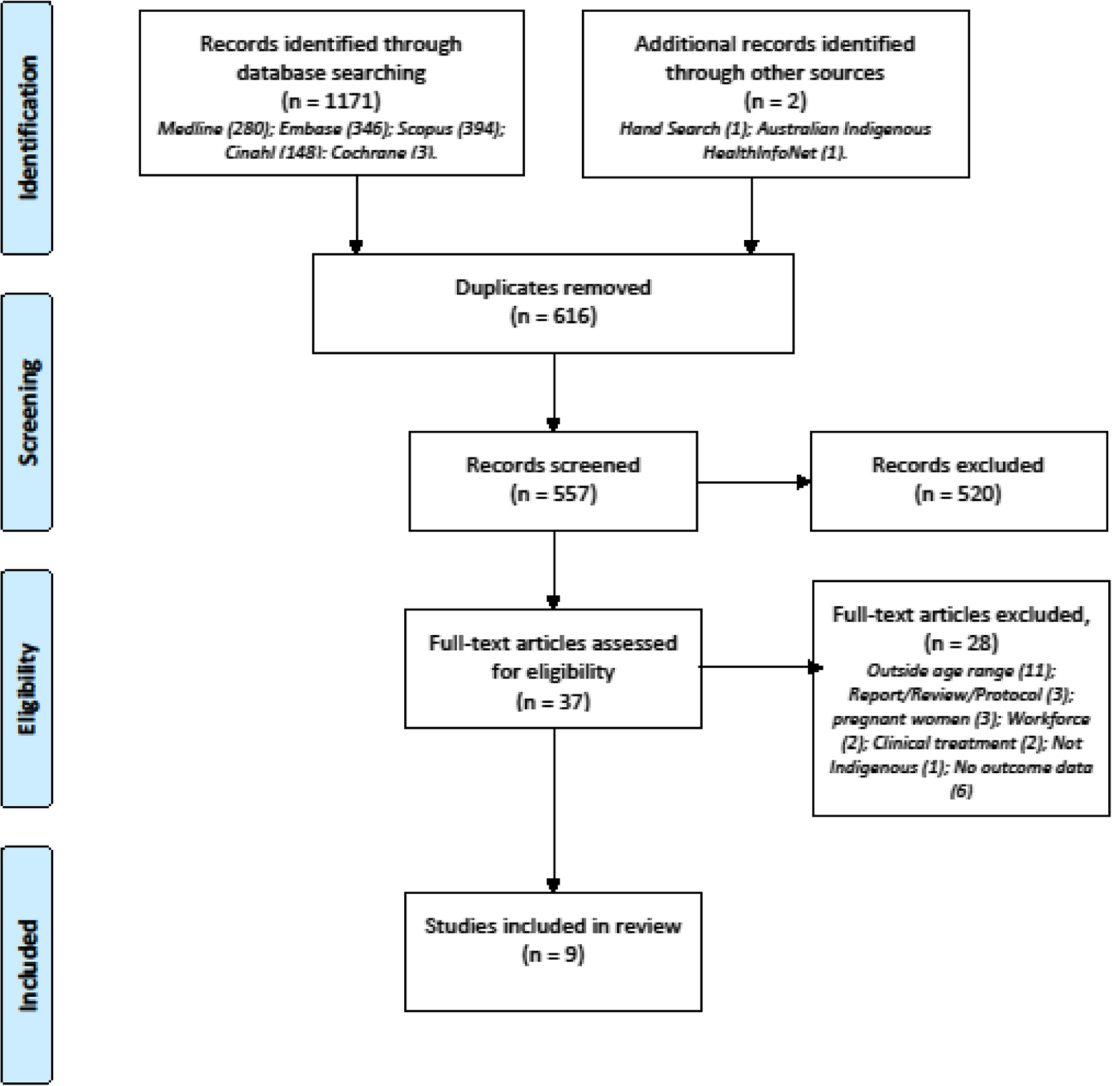
PRISMA study selection flow chart

The initial search yielded 1173 records, 616 duplicates were removed, and 557 records remained. Of these 520 were excluded as they were either: descriptive or qualitative; included a population that was outside, or did not include, the criterion age range; maternal interventions; prevalence studies; or workforce related. The search of the grey literature yielded four articles for review of which none met the inclusion or exclusion criteria. Hand searching yielded one article. Thirty-seven eligible articles remained of which 9 met the inclusion or exclusion criteria for this review. Figure 1 demonstrates study selection.

Table 1 summarises the quality assessment, design, population, interventions, ecological approach and community engagement intensity and outcomes of each study.

**Table 1:** Description of studies included in systematic review (n = 9) by study, study design, participants, community engagement, ecological approach, intervention, outcomes and quality assessment rating

Study (n = 9)	Study design	Population Geographical area	Participants			EAS score CEI	INTERVENTION Strategies Targets [31] Setting Timeframe	Outcomes				Quality Assessment EPHPP Global Score
			Gender	Age	Number			DMFT/DMFS	Caries	Knowledge and/ or behaviour	Other	
**Arantes et al, 2010** [37]	Repeat cross-sectional study (nested in prevalence study).	Xavante people of Brazil	Both (results not reported by gender)	≥2 years and 11–15 years	DMFT outcome (11–15 years):	3	STRATEGY(S) *n* = 3: education; prevention (Fluoride); and clinical. Using principles of participation of the community, promotion of general health, personnel training, utilisation of appropriate technology and fluoride	Outcome: DMFT/DMFS score	↓ in incidence of caries among 11–15 years from 80 to 53% between 1999 and 2007 (nt)			Weak
						Moderate						
		Oe village only - Etenheritipá			Time 1 (1999) = 212 (29)							
								Mean DMFS score for				
					Time 2 (2004) = 281 (64)		TARGET(S): IND	11–15-year olds fell from 4.95 in 2004 to 2.39 in 2007				
							SETTING(S): Community and clinical (clinical implied)					
					DMFS outcome (11-15 years): Time 1 (2004) = 281 (64)							
							TIMEFRAME: 8 years					
								(p < 0.01)				
					Time 2 (2007) = 372 (66)							
**Carberry 2004** [38]	Pre-Post design	American Indian Navajo people	Both (results not reported by gender)	3–13 years	Time 1 = 180	0	STRATEGY(S): Fluoride (0.2%) rinsing program (2 rinses per week)	↓ DMFS score to 0.8 for 11 year old children (nt)			Compared with one year previous:	Weak
					Time 2 = 251	Moderate						
		Rural					TARGET(S): IND and INT (family)				↑25% in dental appointments (nt)	
							SETTING(S): Home and School					
							Home: 3–4 years of age participated in the ‘Headstart’ program				↓% in dmft of 2nd year ‘Headstart’ children from 16.3 to 7.7% (nt)	
							School: 5–13 years of age					
							TIMEFRAME: 1 year					
											↑'Evidence’ of dental care by 1/3rd: to 67% in school children and to 47% in the ‘HeadStart’ children (nt)	
											↓% of ‘active decay’ from 63 to 37% (nt)	
											Rate of sealant applications doubled	
											↑ in crowns on primary teeth 5 vs 49	
											↑ in pulpotomy procedures 15 vs 42	
**Chen, et al, 2011** [39]	Pre-Post design	Truku children in the Chongguang Tribe (Taiwan) Rural (Wenlan Village, Xiulin Township)	m = 34; f = 33	3–15 years	TOTAL: *n* = 67	2	STRATEGY(S) n = 5: lectures for children and parents; teaching videos for children; teaching children how to brush their teeth correctly; giving out tooth cleaning supplies; and handing out prepared dental care manuals to children			Outcome: Dental care knowledge	Outcome:	Weak
					7–15 years = 56	Light					Dental plaque levels	
										√ ↑ in tooth-cleaning habits; knowledge of caries; knowledge of change of teeth; and periodical examination schedule	(in subset of children *n* = 16)	
											√ ↓in dental plaque	
							TARGET(S): IND (including as member of peer group at school) and INT (family)					
							SETTING(S): not described (implied either community OR school)					
										(p < 0.001)		
							TIMEFRAME: 1 year			√ ↑ in dietary habits		
**Harrison et al, 2006** [40]	Repeat cross-sectional	Canadian First Nations people Remote	Both (results not reported by gender)	All children on reserve (age not specified)	Time 1 = 34	3	STRATEGY(S) n = 4: daily school ‘brush-ins’; weekly fluoride rinse for children ≥9 years and tri-annual fluoride varnish applications < 9 years; incentives; and anticipatory guidance for parents, classroom health education				Outcome: ‘Time units’ needed to complete dental treatment for children	Weak
					Time 2 = 49	Moderate						
											Reduction in hours required to restore (p ≤ 0.001) or extract (p ≤ 0.01) teeth and to engage in preventative therapy (p ≤ 0.001)	
							TARGET(S): IND (including as member of peer group at school) and INT (family)					
							SETTING(S): School and clinic					
							TIMEFRAME: 3 years					
**Johnson et al, 2014** [41]	Repeat cross-sectional	Australian Aboriginal and/or Torres Strait Islander people	Both (53% male)	6–15 years	TOTAL: *n* = 324	0	STRATEGY: Introduction of a reticulated fluoridated water supply	Outcome:	Outcome: caries (primary and permanent dentition)		Fewer teeth had restorations in both surveys	Moderate
					10–12 years:	Light		dmft and DMFT				
					Time 1 = 131 Time 2 = 67 (dmft and caries in primary dentition only)		TARGET(S): Community	√ ↓ mean dmft (missing & filled only) at 10 years				
							SETTING(S): Environmental					
									↓ in overall caries prevalence and severity from 2005 to 2012 by 37.3%.			
		Remote (5 small communities North Queensland)					TIMEFRAME: 7 years					
								(p < 0.05)				
					10–15 years:			√ ↓ mean: DMFT at 15 years				
					Time 1 = 224 Time 2 = 127 (DMFT and caries in permanent dentition only)							
								Decayed at 15 years				
								Missing at 14 years				
								Filled at 10–15 years				
								(p < 0.05)				
**McNab et al 2008** [42]	Pre-Post design	Canadian First Nations people	Both (results not reported by gender)	5–16 years	Time 1 = 26 Time 2 = 40	2	STRATEGY(S) n = 4: daily brush-ins; fluoride application; educational presentations; and incentive scheme	Outcome: dmfs/DMFS and dmft/DMFT			Prior to intervention 8% children cavity free	Weak
						Moderate						
		Remote			13 participated in pre and post intervention evaluation						Post intervention 32% cavity free	
							TARGET(S): IND including as member of peer group at school through the education strategy	√ ↓ dmfs/DMFS				
								(*p* < 0.005)				
								√ ↓ dmft/DMFT				
					Numbers within age range not stated		SETTING(S): School	(p < 0.05)				
							TIMEFRAME: 3 years					
**Olubunmi & Olushola, 2002** [43]	Randomised Controlled Trial	Nigeria	TOTAL: m = 59; f = 61	11–12 years	TOTAL: *n* = 120	2	STRATEGY(S): Health Education comparing three groups (two intervention and one control)				Outcome: Oral hygiene, debris and calculus scores	Strong
		Urban			Intervention = 8 (2 groups of 40)	Light						
			Grp1: m = 22; f = 18									
							Intervention 1: 20 min oral health education video of a story acted by well-known local actors Intervention 2: 20 min verbal oral health education				Post intervention all scores lower	
			Grp 2: m = 19; f = 21									
											√ differences in mean debris scores between intervention and control groups with lowest score for verbal education (p < 0.001)	
			Grp 3: m = 18; f = 22									
							TARGET(S): IND (peer group at school).					
							SETTING(S): School					
							TIMEFRAME: 6 weeks					
											√ differences in mean calculus scores between intervention and control groups	
											(p < 0.001)	
											√ differences in oral hygiene scores between intervention and control groups	
											(p < 0.001)	
											Video education showed greater odds of improvement in oral hygiene than verbal education video	
**Wilder et al 2014** [36]	Pre-Post (cohort) study (nested within a Mixed methods design)	Australian Aboriginal and Torres Strait Islander people	m = 7; f = 10	5–12 years (mean age 7.5 years)	TOTAL: *n* = 17	4	STRATEGY(S) *n* = 5. Pilot study of ‘New model’ of care consisting of 5 intervention strategies delivered monthly to children and families in the child’s home. Strategies: partnerships (including community consultations); employment of Aboriginal and/or Torres Strait Islander health workers); ‘cultural aides and equipment’ (timers, charts, toothbrushes); education package; and oral health assessment and dental treatment	Outcome: dmfs			Outcomes: Dental and periodontal indicators	Strong
					Numbers within age range not stated.	Moderate		↓ dmfs from 3.7 to 3.5 (nt)				
											√ ↓ in proportion of unmet restorative needs compared to baseline 71% vs 34.4%	
		Rural.										
											(p < 0.05)	
											√ ↑in average numbers of fissure sealants present in permanent teeth from 0.4 to 1.6	
							TARGET(S): IND and INT (families).					
											(p < 0.01)	
											Gingival Index change: 58.8% no change; 23.5% level 1 improvement; 5.9% level 2 improvement, and less level 1 and 2 dis-improvement	
							SETTING(S): Home, school and community					
							TIMEFRAME: 10 months					
											Plaque Index change: 47.1% no change; 29.4% level 1 improvement; 5.9% level 2 improvement; 17.6% level 1 of dis-improvement	
**Yang et al, 2009** [44]	Cluster randomised controlled trial	Taiwan (Pingtung County)	TOTAL: m = 68; f = 67.	7th Grade	TOTAL: *n* = 135 Intervention = 607	0	STRATEGY(S): Intervention group received a specially designed education program covering a range of oral health-related topics delivered using 8 modules (40 min each held once per week)			Outcome: Knowledge and Behaviour	Most (87%) students considered the educational program excellent or good	Moderate
						Light						
		Rural	Intervention: m = 33; f = 34.									
										√ ↑ oral health knowledge		
							TARGET(S): IND (as member of peer group at school)			(p < 0.001)		
										√ ↑ increase in tooth-brushing frequency (p < 0.001)		
							SETTING(S): School setting					
							TIMEFRAME: 8 weeks					
										√ ↓ in tobacco use (*p* = 001)		

**CEI = Community Engagement Intensity**

**EAS = Ecological Approach Score (4 = intervention reported as including at least 2 strategy types and ≥ 3 settings, with lesser scores reflect fewer strategy types and settings, and 0 = 1 strategy regardless of number of settings**

**IND = individual; INT = Interpersonal environment**

**dmft = Number of decayed, missing or filled teeth (primary dentition)**

**dmfs = Number of decayed, missing or filled teeth surfaces (primary dentition)**

**dmft/DMFT = Number of decayed, missing or filled teeth (primary/permanent dentition)**

**DMFT = Number of decayed, missing or filled teeth (permanent dentition)**

**DMFS = Number of decayed, missing or filled surfaces (permanent dentition)**

**↑ = increase; ↓ = decrease**

**√ = statistically significant**

**nt = no test for difference applied**

### Quality assessment

Two of the nine studies in this review were given an EPHPP Global Rating of strong [43, 44]; two as moderate [38, 41], and the remaining five as weak [37, 39, 40, 42, 45]. Additional file 1 describes the quality assessment components of each study. All but one of the included studies [39] were rated either moderate or strong for selection bias. The EPHPP tool defines a study as STRONG for selection bias (score = 1) where it is ‘very likely’ that study participants were representative of the target population AND that there is greater than 80% participation from that population [46]. Scores for selection bias increase the less likely it is that participants are representative of the target population. The majority of studies were rated moderate [37–39, 42, 43] or strong [41, 44] for study design.

### Study design

Study designs included three repeat cross-sectional studies [38, 40, 45] with one of these nested in a prevalence study [40]; four pre-post studies [37, 39, 42, 43] with one nested in a mixed methods study [43]; one randomized controlled trial (RCT) [44], and one cluster RCT [41] (Table 1). Due to the relatively small number, and heterogeneity, of the studies, no meta-analysis was performed.

### Study population

Studies were conducted in diverse countries and geographical areas. Two studies were conducted in Australian rural or remote communities [38, 43]; two studies in Taiwanese rural communities [37, 41]; two in remote Canadian First Nations communities [39, 45]; one in an American Indian (rural) setting [42], one in rural Brazil [40]; and one in urban African (Nigerian) [44] communities (Table 1).

The sample size of studies varied between 17 and 324 participants. Where gender was reported (*n* = 5) there was an even proportion of male and female participants. Five studies reported on outcomes for adolescents [38, 40–42, 44], with two of these studies designed specifically for the age range included in our criterion ie 10–19 years [41, 44] (Table 1). The remainder reported results at a population level and did not specify results for participants in this age range.

### Interventions

Eight out of the nine studies described intervention strategies targeting the individual [37, 39–45]; four of these included the family [37, 42, 43, 45] as the enablers of change in the oral health status of their child (Table 1). This is important in this context as family and peer group (such as in the school setting) are considered part of the child’s interpersonal environment, and can be enablers for strategies targeting the individual [35]. The remaining study reported on an environmental intervention [38] which was the provision of a water reticulation system including fluoride. Schools were the most common setting for study interventions. Three interventions were delivered only in schools [39, 41, 44]; three included schools as one of a number of intervention settings [42, 43, 45]; and one implying that a school was the setting [37].

Five intervention strategies emerged from the review. These included: 1) Educational (*n* = 7) [37, 39–41, 43–45] which targeted behaviour and knowledge of children and/or parents; 2) Clinical (*n* = 4) [39, 40, 42, 43] which included fluoride varnish or rinse and dental treatment; 3) Provision of incentives (*n* = 2), one using cash [45] and the other using ‘prizes’ (no details provided) [39]; 4) Employment of local Health Workers (n = 2) [40, 43]; and 5) Reticulated fluoridated water supply (*n* = 1) [38]. Five studies delivered more than one intervention strategy [37, 39, 40, 43, 45].

### Ecological approach and community engagement intensity

**Fig. 2:**
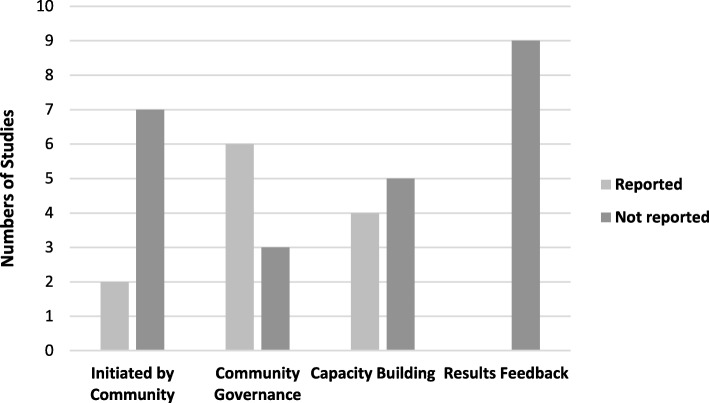
Key Features of Indigenous Community Engagement reported in studies (*n* = 9)

Only one study [43] was given an EAS score of four; indicating it included at least two strategy types and was implemented in more than three settings. Furthermore, no study reported a strong level of CEI. These were concerning results given that the features of these measures are recommended for conduct of research in Indigenous communities. When CEI features were examined six studies reported community governance or engagement in the research however little information on the nature of this was provided. Five studies reported that ‘capacity building’ occurred however when this feature was further examined capacity building was largely the formation of partnerships [39, 40, 42, 43, 45], with no studies describing career development pathways for Indigenous staff, and only one reporting on the participation of the community in decision making [42]. No studies reported providing feedback of results to the participating communities. The number of studies which reported on each key feature of CEI are presented in Fig. 2. Additionally, details of key feature of CEI can be found in Additional File 2. It should be noted that not all studies may have reported details of community engagement despite this being a key component of study design with Indigenous communities.

### Outcomes

Eight of the nine studies reported statistically significant improvements in at least one component of oral health (Table 1). The most frequently reported outcome (*n* = 5) was change in dmft/DMFT or the number of decayed, missing or filled tooth surfaces (dmfs/DMFS) [38–40, 42, 43]; with two of these studies finding significant improvements of between *p* < 0.001 and *p* < 005 [38, 39]. Two studies reported significant changes in oral health knowledge and/or behaviour [37, 41]. Two studies reported a decline in caries prevalence; however, this was not tested for statistical significance [37, 41]. Six studies reported more than one outcome [37–40, 42–44]. A number of other statistically significant outcomes were reported across three studies including: reduction in treatment hours required (p < 0.001) [45]; reduced levels of debris, calculus and oral hygiene scores following video education compared to verbal education (*p* < 0.05) [44]; decreased levels of unmet restorative needs and increased numbers of fissure sealants (*p* < 0.01) [43].

## Discussion

This review of the international peer-reviewed literature which examined interventions aiming to improve the oral health of Indigenous adolescents, found few studies on the topic. While this is unsurprising, it confirms that little is known on effectively engaging with Indigenous adolescents to improve oral health.

In most countries, a large proportion of the Indigenous population now live in urban areas [47]; however only one study was conducted in an urban setting. The lack of studies targeting Indigenous children and adolescents has been found in other systematic reviews [34, 48] and confirms the need to explore strategies to effectively engage this population.

No study was assessed as reporting a strong community engagement intensity and only one study was given an EAS score of four representing a strong ecological approach to intervention design [43]. Despite majority of the studies reporting a significant improvement in oral health, the overall absence of Indigenous community engagement and governance of the research indicates that the researchers did not partner equitably or consider that participating communities should take a leadership role. This raises concerns regarding the sustainability, scalability and long-term impact of interventions which show promise. Engagement in and leadership of all research conducted in their communities is central to improving Indigenous health (including oral health) [48, 49]. Guidelines recommending this approach exist across many countries for example in Australia [29], New Zealand [50] and Canada [51]. As communities emerge from the colonial era Indigenous methodologies are increasingly being described and applied [52]. Including strong capacity building strategies that create pathways for leadership and employment is essential to the successful implementation of research in Indigenous communities, however this was clearly lacking in all studies reviewed [49]. The voices of Indigenous adolescents were absent in all studies, and must be included to ensure relevant design and successful implementation of all aspects of the research including the interpretation of results [53].

Adopting participatory action-based research (PABR) methods including co-design will enable adolescents to engage in and guide all aspects of program design, implementation and completion [54]. PABR has been shown to be highly effective in social and health research with adolescents and Indigenous communities [54], and brings adolescents and researchers together to explore and then co-design interventions [55]. Few studies applied an Ecological (or multi systems) model [35], an approach becoming widely accepted as necessary in addressing delivery of public health interventions such as community based oral health programs [15]. This systematic review highlights that the voices of Indigenous adolescents have not been included in the co-design of community-based oral health programs that foster local leadership and build community capacity in order to improve the oral health of this population, particularly in Australia.

Only one study achieved promising results across several of the quality assessment component ratings [43]. This study received a strong EPHPP Global quality assessment score (one of only two studies assessed as such); the highest EAS; statistically significant improvements in two measures of oral health; and a moderate level of CEI. Whilst the sample size of this Australian study was small and the target age range was children aged 5–12 years, the intervention demonstrates a successful multi strategy (*n* = 5) approach that may be adopted in the design of other community-based oral health programs for Australian Aboriginal and Torres Strait Islander adolescents. These strategies are described in Table 1 and include: partnerships; employment of Aboriginal and/or Torres Strait Islander health workers; ‘cultural aides and equipment’; an education package; and oral health assessment and dental treatment.

The results from this review contributed to a workshop facilitated by the authors which included representatives from various Australian and New South Wales (NSW) based organisations involved in the delivery of health services and Aboriginal and Torres Strait Islander research and vocational education, along with Aboriginal and Torres Strait Islander adolescents from various communities across NSW. The purpose was to collaborate and discuss potential strategies to co-design an oral health program with, and for Aboriginal and Torres Strait Islander adolescents. An outcome of this workshop was the agreement to establish an Aboriginal Youth Advisory Group that will guide the development of an oral health program for adolescents.

### Limitations

A limitation of this systematic review is that many studies were small and therefore results must be interpreted with caution. Another limitation is that several studies included an age range wider than that of 10–19 years. However, this does not detract from the findings that there are limited effective and culturally competent oral health programs targeting Indigenous adolescents and none that incorporate the voices of this population into the design of the program.

## Conclusion

This review found very few good quality peer reviewed international studies of community-based oral health interventions which address the complex and diverse needs of Indigenous adolescents. The absence of Indigenous community engagement and of the voices of Indigenous adolescents was notable and raises questions about the cultural competence and long-term scalability, sustainability and effectiveness of the interventions included in this review. Community based oral health programs targeting Indigenous adolescents must include strong Indigenous community leadership and governance at all stages of the research including design; adopt participatory action-based research approaches and apply an ecological model.

## Supplementary information

**Additional file 1.** Description of EPHPP Quality Assessment Component Ratings by study. All components of EPHPP Quality Assessment tool described by study

**Additional file 2.** Key Features of Indigenous community initiation of the research, governance, engagement and/or capacity building. Proportion of included studies that described the key features of Community Engagement including community initiation of the research, governance, engagement and/or capacity building

## Data Availability

The datasets used and/or analysed during the current study are available from the corresponding author on reasonable request.

## Abbreviations

CEI: Community Engagement Intensity
dmfs/DMFS: The number of decayed, missing or filled tooth surfaces
dmft/DMFT: The number of decayed, missing and filled primary and/or permanent teeth
EAS: Ecological Approach Score
EPHPP: Quality Assessment Tool for Quantitative Studies from the McMaster University Effective Public Health Practice Project
NSW: New South Wales
PABR: Participatory Action-Based Research
RCT: Randomized Controlled Trial
WHO: World Health Organisation

## References

[1] World Health Organization. Health Topics: Indigenous Populations 2018 [Available from: https://www.who.int/topics/health_services_indigenous/en/.

[2] Stephens C, Nettleton C, Porter J, Willis R, CLark S (2005). Indigenous peoples' health—why are they behind everyone, everywhere?. Lancet.

[3] Tiwari L, Jamieson L, Broughton J, Lawrence HP, Batliner TS, Arantes R (2018). Reducing indigenous Oral health inequalities: a review from 5 nations. J Dent Res.

[4] Williams S, Jamieson L, MacRae A, Gray C (2011). Review of indigenous oral health. Indigenous HealthBulletin.

[5] Alves Filho P, Santos RV, Vettore MV (2013). Social and environmental inequities in dental caries among indigenous population in Brazil: evidence from 2000 to 2007. Rev..

[6] Levin A, Sokal-Gutierrez K, Hargrave A, Funsch E, Hoeft KS. Maintaining traditions: A qualitative study of early childhood caries risk and protective factors in an indigenous community. International Journal of Environmental Research and Public Health. 2017;14 (8) (no pagination)(907). 10.3390/ijerph14080907 PMC5580610 28800116

[7] Lawrence HP, Cidro J, Isaac-Mann S, Peressini S, Maar M, Schroth RJ (2016). Racism and Oral health outcomes among pregnant Canadian aboriginal women. J Health Care Poor Underserved.

[8] Shekhawat KS, Chauhan A, Nordstroem M (2016). Dental pain and its impact on quality of life among indigenous adolescents of Himalayas (Ladakh). India Indian J Dent Res.

[9] Silveira MF, Freire RS, Nepomuceno MO, Martins AM, Marcopito LF (2015). Tooth decay and associated factors among adolescents in the north of the state of Minas Gerais, Brazil: a hierarchical analysis. Ciencia & saude coletiva.

[10] Silveira MF, Freire RS, Nepomuceno MO, Martins AM, Marcopito LF (2016). Severity of malocclusion in adolescents: populational-based study in the north of Minas Gerais. Brazil Rev Saude Publica.

[11] Banderas JA, Toshikasu O, Gonzalez M (1999). Oral mucosa lesions in Mazahua Indian adolescents. Acta Odontol Latinoam.

[12] Centers for Disease C, Prevention. Dental caries in rural Alaska Native children--Alaska, 2008. MMWR Morb Mortal Wkly Rep. 2011;60(37):1275–8. 21937973

[13] Jamieson LM, Koopu PI (2006). Exploring factors that influence child use of dental services and toothbrushing in New Zealand. Community Dent Oral Epidemiol.

[14] Jayashantha P, Johnson NW (2016). Oral health status of the Veddas--Sri Lankan indigenous people. J Health Care Poor Underserved.

[15] Tsai C, Blinkhorn A, Irving M (2017). Oral health Programmes in indigenous communities worldwide-lessons learned from the field: a qualitative systematic review. Community Dent Oral Epidemiol.

[16] Chi DL (2013). Reducing Alaska native paediatric oral health disparities: a systematic review of oral health interventions and a case study on multilevel strategies to reduce sugar-sweetened beverage intake. Int J Circumpolar Health.

[17] Patel J, Durey A, Hearn L, Slack-Smith LM (2017). Oral health interventions in Australian aboriginal communities: a review of the literature. Aust Dent J.

[18] Australian Bureau of Statistics. Census of Population and Housing: Reflecting Australia - Stories from the Census 2016 [Available from: http://www.abs.gov.au/ausstats/abs@.nsf/Lookup/by%20Subject/2071.0~2016~Main%20Features~Aboriginal%20and%20Torres%20Strait%20Islander%20Population%20Data%20Summary~10.

[19] Azzopardi PS, Kennedy EC, Patton GC, Power R, Roseby RD, Sawyer SM (2013). The quality of health research for young indigenous Australians: systematic review. Med J Aust.

[20] Australian Institute of Health and Welfare. Aboriginal and Torres Strait Islander Health Performance Framework Canberra2017 [Available from: https://www.aihw.gov.au/reports/indigenous-health-welfare/health-performance-framework/contents/tier-1/1-11.

[21] Arain M, Haque M, Johal L, Mathur P, Nel W, Rais A (2013). Maturation of the adolescent brain. Neuropsychiatr Dis Treat.

[22] Australian Institute of Health and Welfare. Aboriginal and Torres Strait Islander adolescent and youth health and wellbeing 2018: in brief. Cat. no. IHW 198. Canberra: AIHW; 2018.

[23] Organization WH. Adolescents’ health-related behaviours: World Health Organization; 2014 [Available from: https://apps.who.int/adolescent/second-decade/section4.

[24] Toombs M (2013). Indigenous Australians and health: the wombat in the room.

[25] Organisation WH. Adolescent Health 2018 [Available from: http://www.who.int/topics/adolescent_health/en/.

[26] PRISMA. Preferred Reporting Items for Systematic Reviews and Meta-Analyses (PRISMA) website 2015 [Available from: http://www.prisma-statement.org/. 10.1188/15.ONF.552-554 26302284

[27] National Collaborating Centre for Methods and Tools. Quality Assessment Tool for Quantitative Studies. Hamilton, ON: McMaster University. (Updated 13 April, 2010) 2008 [Available from: http://www.nccmt.ca/resources/search/14.

[28] Liberato S, Brimblecombe J, Ritchie J, Ferguson M, Coveney J. Measuring capacity building in communities: a review of the literature. BMC Public Health. 2011;11. 10.1186/1471-2458-11-850 PMC3229539 22067213

[29] National Health and Medical Research Council. The NHMRC Road Map II: A strategic framework for improving the health of Aboriginal and Torres Strait Islander people through research. Canberra, Australia: National Health and Medical Research Council. ; 2010 [Available from: http://www.nhmrc.gov.au/guidelines/publications/r47 . 10.5694/j.1326-5377.2008.tb01767.x 18459924

[30] Gwynn J, Lock M, Turner N, Dennison R, Coleman C, Kelly B, et al. Aboriginal and Torres Strait islander community governance of health research: turning principles into practice. Aust J of Rural Health. 2015. 10.1111/ajr.12182 25823497

[31] Black A (2007). Evidence of effective interventions to improve the social and environmental factors impacting on health: informing the development of indigenous community agreements Canberra.

[32] National Health and Medical Research Council. Keeping research on track: A guide for Aboriginal and Torres Strait Islander peoples about health research ethics. Canberra (Australia): National Health and Medical Research Council 2005 [Available from: http://www.nhmrc.gov.au/guidelines/publications/e65.

[33] National Health and Medical Research Council. Values and Ethics: Guidelines for ethical conduct in Aboriginal and Torres Strait Islander Research. Canberra (Australia): National Health and Medical Research Council. ; 2003 [Available from: http://www.nhmrc.gov.au/guidelines/publications/e52.

[34] Gwynn J, Sim K, Searle T, Senior A, Lee A, Brimblecombe J. Effect of nutrition interventions on diet related and health outcomes of aboriginal and Torres Strait islander Australians: a systematic review. BMJ Open. 2018. 10.1136/bmjopen-2018-025291 PMC6500365 30948579

[35] Cargo M, Marks E, Brimblecombe JK, Scarlett M, Maypilama E, Dhumkay J (2011). Integrating an ecological approach into an aboriginal community-based chronic disease prevention program: a longitudinal process evaluation. BMC Public Health.

[36] Richard L, Gauvin L, Raine K (2011). Ecological models revisited: their uses and evolution in health promotion over two decades. Annu Rev Public Health.

[37] Chen CC, Huang HK, Huang MJ, Wu CH (2011). Educational intervention can improve dental care knowledge in aboriginal tribal children. Tzu Chi Medical Journal.

[38] Johnson NW, Lalloo R, Kroon J, Fernando S, Tut O (2014). Effectiveness of water fluoridation in caries reduction in a remote indigenous community in far North Queensland. Aust Dent J.

[39] Macnab AJ, Rozmus J, Benton D, Gagnon FA (2008). 3-year results of a collaborative school-based oral health program in a remote first nations community. Rural Remote Health.

[40] Arantes R, Santos RV, Frazao P (2010). Oral health in transition: the case of indigenous peoples from Brazil. Int Dent J.

[41] Yang YH, Sue RL, Warnakulasuriya S, Dasanayake AP (2009). Promoting better oral health practices among aboriginal Taiwanese adolescents: a school based oral health education intervention program. J Health Care Poor Underserved.

[42] Carberry F, Cloud B, Finster C. Dental Disease Control in Pine Hill, New Mexico. New York State Dental Journal. 2004;70(2). 15124338

[43] Wilder S, Nelson J, Morgan M, Mariño R. ‘Indigie-Grins’: an Indigenous youth oral health research project. Australian Indigenous Health Bulletin. 2014;14(2).

[44] Olubunmi B, Olushola I (2013). Effects of information dissemination using video of indigenous language on 11-12 years children's dental health. Ethiop..

[45] Harrison RL, MacNab AJ, Duffy DJ, Benton DH (2006). Brighter smiles: service learning, inter-professional collaboration and health promotion in a first nations community. Can J Public Health.

[46] MacMaster University (2009). Quality assessment tool for quantitative studies dictionary.

[47] United Nations. Urban Indigenous Peoples and Migration: Challenges and Opportunities United Nations,; n.d [Available from: https://www.un.org/esa/socdev/unpfii/documents/6_session_factsheet2.pdf.

[48] Gwynne K, Jeffries L, Lincoln M. Health care outcomes for aboriginal Australians: a systematic review. Aust Health Rev. 2016. 10.1071/AH17142 29335090

[49] National Health and Medical Research Council. Ethical conduct in research with Aboriginal and Torres Strait Islander Peoples and communities: Guidelines for researchers and stakeholders. In: Commonwealth of Australia, editor. Canberra2018.

[50] Health Research Council of New Zealand. Guidelines for researchers on health research involving Maori 2010 [Available from: http://www.hrc.govt.nz/sites/default/files/Guidelines%20for%20HR%20on%20Maori-%20Jul10%20revised%20for%20Te%20Ara%20Tika%20v2%20FINAL%5B1%5D.pdf.

[51] Canadian Institutes of Health Research NSaERCoC, & Social Sciences and Humanities Research Council of Canada, . Tri-council policy statement 2: Ethical conduct for research involving humans. 2014 [Available from: http://www.pre.ethics.gc.ca/pdf/eng/tcps2/TCPS_2_FINAL_Web.pdf.

[52] Wilson S (2008). Research is ceremony: indigenous research methods.

[53] Vandermorris A, Bhutta ZA (2017). How Canada can help global adolescent health mature. Reprod Health.

[54] Fleming JBT. Involving children and young people in health and social care research. London: Routledge. 2012.

[55] Hagen P CP, Metcalf A, et al. , editor Participatory Design of evidence-based online youth mental health promotion, prevention, early intervention and treatment. Young and Well CRC 2012; Melbourne.

