# Supplementary material for: Community based programs to improve the oral health of Australian Indigenous adolescents: a systematic review and recommendations to guide future strategies
Source: BMC Health Serv Res. 2020 May 6;20:384. doi: 10.1186/s12913-020-05247-w (PMC7204065; doi:10.1186/s12913-020-05247-w)
Supplement: Supplementary file 1 — Additional file 1. Description of EPHPP Quality Assessment Component Ratings by study. All components of EPHPP Quality Assessment tool described by study [file 12913_2020_5247_MOESM1_ESM.docx]

| **Study** | | **Study Design** | **Author Assessor** | **EPHPP Quality Assessment Component Ratings** | | | | | | **EPHPP Final Global Score^a^** |
| --- | --- | --- | --- | --- | --- | --- | --- | --- | --- | --- |
| **Author** | **Year** |  |  | **Selection Bias** | **Study Design** | **Confounders** | **Blinding** | **Data Collection Methods** | **Withdrawals & Drop-outs** |  |
| Arantes et al^43^ | 2010 | Repeat Cross-sectional^b^ | AM | Moderate | Weak | N/A | Moderate | Moderate | Weak | Weak |
|  |  |  | JS | Moderate | Weak | N/A | Moderate | Moderate | Weak | Weak |
| Carberry et al^40^ | 2004 | Pre-Post (cohort) design | AM | Moderate | Moderate | N/A | Weak | Weak | Weak | Weak |
|  |  |  | JG | Moderate | Moderate | N/A | Weak | Weak | Weak | Weak |
| Chen et al^42^ | 2011 | Pre-Post (cohort) design | AM | Strong | Moderate | N/A | Weak | Strong | Weak | Weak |
|  |  |  | JS | Strong | Moderate | N/A | Weak | Strong | Weak | Weak |
| Harrison et al^44^ | 2006 | Repeat Cross-sectional | AM | Strong | Weak | Weak | Strong | Moderate | Weak | Weak |
|  |  |  | JS | Strong | Weak | Weak | Strong | Moderate | Weak | Weak |
| Johnson et al^39^ | 2014 | Repeat Cross-sectional | AM | Moderate | Weak | N/A | moderate | Strong | Strong | Moderate |
|  |  |  | JS | Moderate | Weak | N/A | Moderate | Strong | Strong | Moderate |
| Macnab et al^41^ | 2008 | Pre-Post (cohort) design | AM | Weak | Moderate | N/A | Moderate | Weak | Strong | Weak |
|  |  |  | JG | Weak | Moderate | N/A | Moderate | Weak | Strong | Weak |
| Olumbuni & Olumhola^37^ | 2002 | RCT | AM | Strong | Strong | Strong | Strong | Strong | Strong | Strong |
|  |  |  | JS | Strong | Strong | Strong | Strong | Strong | Strong | Strong |
| Wilder et al^36^ | 2014 | Pre-Post (cohort) design^c^ | AM | Moderate | Moderate | N/A | Moderate | Moderate | Strong | Strong |
|  |  |  | JG | Moderate | Moderate | N/A | Moderate | Moderate | Strong | Strong |
| Yang et al^38^ | 2009 | Cluster RCT | AM | Strong | Strong | Strong | Moderate | Moderate | Weak | Moderate |
|  |  |  | JS | Strong | Strong | Strong | Moderate | Moderate | Weak | Moderate |
| ^a^ If component score = N/A then that component not included in final global score; ^b^ nested in prevalence study; ^c^ nested in mixed methods study. | | | | | | | | | | |

Additional File 1: Description of EPHPP Quality Assessment Component Ratings by study
