# Supplementary material for: Community based programs to improve the oral health of Australian Indigenous adolescents: a systematic review and recommendations to guide future strategies
Source: BMC Health Serv Res. 2020 May 6;20:384. doi: 10.1186/s12913-020-05247-w (PMC7204065; doi:10.1186/s12913-020-05247-w)
Supplement: Supplementary file 2 — Additional file 2. Key Features of Indigenous community initiation of the research, governance, engagement and/or capacity building. Proportion of included studies that described the key features of Community Engagement including community initiation of the research, governance, engagement and/or capacity building [file 12913_2020_5247_MOESM2_ESM.docx]

| **Key Feature** | **Proportion and Feature Type (where relevant) Reported** | **Proportion Not Reported** |
| --- | --- | --- |
| 1. Initiated by Aboriginal and Torres Strait Islander community leaders, elders or organisations | 22% ^44, 40^ | 78% ^36, 37, 38, 39, 41, 42, 43^ |
| 1. Community governance, engagement, or other cultural considerations *(primary feature reported only and differing in quality/complexity reported)* | *33% organisational engagement:* ^40, 41, 43^  *44% community members engaged:* ^40, 39, 41, 44^  *11% Not specified:* ^36^ | 33% ^37, 38, 42^ |
| 1. Capacity building^28^ | *22% learning opportunity and skill development:* ^40, 43^  *44% partnership:* ^40, 36, 41, 44^  *11% participatory decision making:* ^40^  *0% career development pathway*  *11 % sense of community: ^40^* | 44% ^37, 38, 39, 42^ |
| 1. Results feedback | *0 % individual feedback*  *0% community/group feedback*  *0 % Aboriginal organisation feedback* | 100% |
| ^†^ The total percentage (reported + not reported) may exceed 100 (other than for Feature 1) as some studies described more than one Feature Type. | | |

**Additional File 2: Reported study *(n=9)* Key Features of Indigenous community initiation of the research, governance, engagement and/or capacity building. ^†^**
